# A narrative review of magnetic resonance imaging findings in pediatric idiopathic intracranial hypertension

**DOI:** 10.1002/hsr2.70111

**Published:** 2024-10-07

**Authors:** Abdolreza Sheibani, Narges Hashemi, Behnam Beizaei, Nahid Tavakkolizadeh, Ahmad Shoja, Neda Karimabadi, Houshang Mirakhorli, Parsa Hasanabadi, Asma Payandeh, Ehsan Hassannejad

**Affiliations:** ^1^ Department of Radiology Golestan Hospital, Ahvaz Jundishapur University of Medical Sciences Ahvaz Iran; ^2^ Department of Pediatrics School of Medicine, Mashhad University of Medical Sciences Mashhad Iran; ^3^ Department of Radiology Faculty of Medicine, Mashhad University of Medical Sciences Mashhad Iran; ^4^ Department of Radiology School of Medicine, Birjand University of Medical Sciences Birjand Iran; ^5^ Pharmacy Faculty Mashhad University of Medical Sciences Mashhad Iran; ^6^ Student Research Committee, Kurdistan University of Medical Sciences Sanandaj Iran; ^7^ Student Committee of Medical Education Development, Education Development Center Kurdistan University of Medical Sciences Sanandaj Iran; ^8^ Medicine Faculty Kurdistan University of Medical Sciences Sanandaj Iran; ^9^ Faculty of Medicine Mashhad University of Medical Sciences Mashhad Iran

**Keywords:** brain MRI, children, imaging, intracranial hypertension

## Abstract

**Background and Aims:**

Idiopathic intracranial hypertension (IIH) is a rare neurological disorder in the pediatric population which is defined as an increase in intracranial pressure (ICP) without the presence of brain parenchymal lesions, hydrocephalus, or central nervous system infection. In this study, we have determined the magnetic resonance imaging (MRI) findings in IIH patients.

**Methods:**

A comprehensive literature search was conducted using the electronic databases including Web of Sciences, Scopus, and Pubmed to identify suitable and relevant articles using keyword search methods. The search included keywords such as “idiopathic intracranial hypertension,” “pseudotumor cerebri,” “MRI,” and “pediatrics.” The search was limited to the available publications up to January 2024.

**Results:**

MRI plays a crucial role in diagnosing IIH by excluding secondary causes and revealing neuroimaging findings associated with elevated ICP. Despite fewer studies in children compared to adults, MRI serves as a cornerstone in identifying traditional neuroradiological markers such as empty sella turcica, posterior globe flattening, optic nerve tortuosity, optic nerve sheath distension, and transverse venous sinus stenosis. Additional subtle markers include increased Meckel's cave length, cerebellar tonsillar herniation, and slit‐like ventricles, although these are less reliable. Diffusion‐weighted imaging does not typically show cerebral ADC value changes indicative of cerebral edema in pediatric IIH.

**Conclusion:**

MRI findings provide valuable non‐invasive diagnostic indicators that facilitate early detection, clinical management, and potential surgical intervention in pediatric IIH. The reliability of these MRI markers underscores their importance in clinical practice.

## 1 INTRODUCTION

Idiopathic intracranial hypertension (IIH), also termed pseudotumor cerebri syndrome, is a neurological disorder that occurs rarely in the pediatric population.^1^ It is defined by an increase in intracranial pressure (ICP) without the presence of any brain parenchymal lesions, hydrocephalus, or central nervous system infection, alongside normal cerebrospinal fluid (CSF) composition and the absence of any secondary cause.^2^

Among the general population, the incidence of IIH in adults is 0.9 in 100,000, but among obese women aged 20–44 years, it increases to 19.3 in 100,000.^3^ In the pediatric population (aged <18 years), the incidence is comparable, albeit slightly lower, at a rate of 0.63–0.90 per 100,000.^4^, ^5^ The prevalence of IIH in adults is notably higher among females and those who are obese. However, in children, this connection fluctuates depending on the child's pubertal status. Similar to adults, there is a pronounced female predominance among pubertal children, whereas prepubertal boys and girls are equally susceptible. Moreover, obesity does not constitute a risk factor for prepubertal children; however, it does for pubertal children.^6^, ^7^, ^8^

The manifestations of pediatric IIH are similar to those seen in adults. These symptoms comprise diminished vision accompanied by visual field deficits, dyschromatopsia, cranial neuropathies (primarily affecting the sixth and seventh cranial nerves, and less frequently the fourth), papilledema, and the lack of other neurological impairments.^9^, ^10^ Papilledema holds great significance as a sign in cases of elevated ICP. However, it is essential to note that some patients with pediatric IIH may not exhibit papilledema.^11^

Neuroimaging is the first step in evaluating papilledema when there is a sufficiently high clinical suspicion for elevated ICP. The preferred modality for this purpose would be an MRI of the brain and orbits. The 2013 revised Friedman criteria 2023 evidence‐based diagnostic criteria by Korsbæk et al.^12^ highlight the necessity of MRI characteristics in diagnosing IIH without papilledema (IIHWOP). Consequently, MR venogram (MRV) is recommended, especially for evaluating transverse sinus venous stenosis, which is part of the diagnostic criteria. MRV is essential for diagnosing dural venous thrombosis, particularly if symptoms appear rapidly, there is a history of recent head trauma, or there are coagulopathic risk factors.^12^, ^13^ It has been proposed that MRV imaging may be deemed unnecessary for cases demonstrating highly typical demographics of IIH or another identified secondary cause of IIH. Conversely, occult dural venous thrombosis can be observed in multiple circumstances, thereby having a substantial impact on the therapeutic approach, which necessitates the inclusion of anticoagulation. Consequently, when patients exhibit potential signs of heightened ICP and papilledema, MRV is typically incorporated into the initial diagnostic assessment.^10^, ^14^, ^15^, ^16^, ^17^

MRI plays a crucial role in IIH by eliminating other potential conditions, identifying secondary causes, and evaluating the severity of the disease. Although there is no established evidence allowing the evaluation of IIH severity using MRI in adults, MRI remains a valuable tool in the diagnostic process. Apart from the diagnostic necessity to exclude secondary causes of elevated ICP via neuroimaging, individuals with IIH commonly present positive neuroimaging findings believed to be a consequence of heightened ICP. Nonetheless, the challenge lies in interpreting the clinical implications of these findings, especially where individuals are not suspected of having heightened ICP, and the discovery is merely incidental.^2^, ^18^

Considering that MRI is a non‐invasive method for assessing IIH in pediatric patients, and some parents are reluctant to subject their children to invasive procedures like lumbar puncture, the MRI results can help diagnose these patients.

IIH is an uncommon medical condition affecting children, with fewer studies conducted in this population compared to adults. The focus of this review is to outline traditional MRI findings linked to IIH, including empty sella (ES), posterior globe flattening, optic nerve tortuosity, optic nerve sheath distension, and transverse venous sinus stenosis.

## 2 METHODS

A comprehensive literature search was conducted using the electronic databases including Web of Sciences, Scopus, and Pubmed to identify suitable and relevant articles using keyword search methods. The search included keywords such as “idiopathic intracranial hypertension,” “pseudotumor cerebri,” “MRI,” and “pediatrics.” The search was limited to the available publications up to January 2024. The references of the retrieved articles were also manually checked.

## 3 EMPTY SELLA

An ES is a condition in which the sella turcica is occupied by CSF, either partially or entirely.^17^ ES can be classified as primary or secondary based on the underlying etiology and related pathologies or disorders.^18^ However, the major factors in the development of primary ES are widely accepted to be the insufficiency or absence of a sellar diaphragm and pituitary involution associated with aging. Among the etiological factors in secondary ES are tumors, hydrocephalus, surgical procedures, radiotherapy, trauma, genetic disorders, and pharmaceutical agents.^19^, ^20^, ^21^ Among the etiological factors in secondary ES are tumors, hydrocephalus, surgical procedures, radiotherapy, trauma, genetic disorders, and pharmaceutical agents.^22^, ^23^

**Figure 1:**
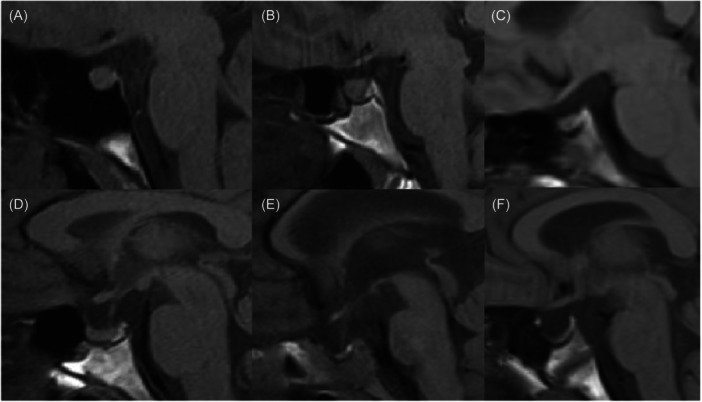
Classification of empty sella (ES) turcica on sagittal T1‐weighted sequence. (A and B) Normal appearance in which the superior aspect of the pituitary gland demonstrates a convex (A) or flat (B) appearance. (C) Mild ES shows a decrease in pituitary height that is less than one‐third of the sella height. (D) Moderate ES, in which the empty space within the Sella turcica occupies a range of one‐third to two‐thirds of its total height. (E) Severe ES, in which the height of the empty space within the sella turcica exceeds two‐thirds of the total height of the sella turcica. (F) Complete ES, in which no observable pituitary parenchyma is detected.

Complete or partial ES turcica can be assessed by utilizing the subsequent reporting classification of the sagittal T1‐weighted sequence^24^ (Figure 1):

- I = normal—the superior aspect of the pituitary gland demonstrates either a convex or flat appearance.
- II = mild—a decrease in pituitary height that is less than one‐third of the sella height.
- III = moderate—the empty space within the sella turcica occupies a range of one‐third to two‐thirds of its total height.
- IV = severe—the height of the empty space within the sella turcica exceeds two‐thirds of the total height of the sella turcica.
- V = complete ES—an enlargement of the sella turcica is present, and no observable pituitary parenchyma is detected.

Studies show ES is observed in 25.9%–78% of pediatric patients with IIH, displaying a diagnostic value marked by low sensitivity and high specificity.^17^, ^25^, ^26^ However, recent evidence‐based diagnostic criteria for IIH have removed ES as an MRI characteristic associated with IIH, indicating a shift in the understanding of its diagnostic relevance.^12^ The etiology of the ES has traditionally been linked to the prolonged impact of high‐pressure pulsatile CSF, causing an arachnocele to herniate downward into the Sella. In addition, there have been theories positing that primary ES may be linked to the enlargement of the Sella turcica, which is caused by prolonged elevation of ICP.^27^, ^28^ It has been demonstrated that prepubescent children may manifest considerably lower frequencies of ES when compared with adolescents and adults.^17^ The lower occurrence of the ES in prepubescent children might be associated with a shorter period of elevated ICP before imaging, as bony remodeling over time is required to develop an ES.^29^, ^30^

## 4 POSTERIOR GLOBE FLATTENING

**Figure 2:**
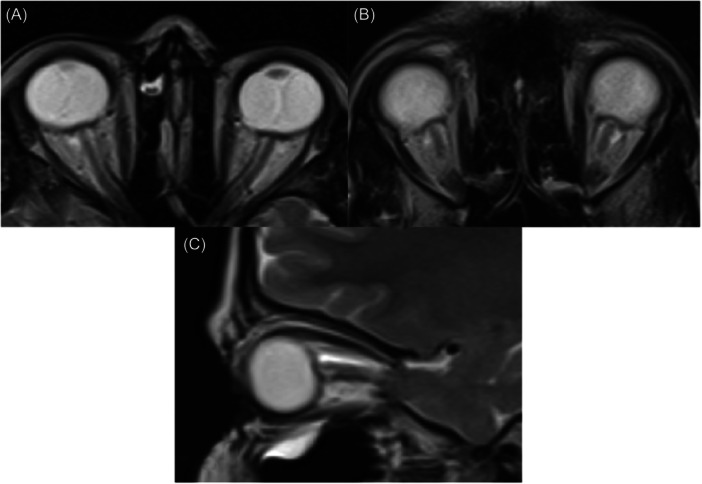
Posterior globe flattening. Axial (A and B) and sagittal (C) T2‐weighted MRI show disturbance of the normal convexity and straightening of the globe's outward curvature at its attachment to the optic nerve. MRI, magnetic resonance imaging.

Disturbance of the normal convexity and straightening of the globe's outward curvature at its attachment to the optic nerve is known as flattening at the posterior aspect of the globe. This can be evaluated using axial or sagittal T2‐weighted scans (Figure 2).^31^, ^32^

The determination and extent of posterior globe flattening can be subjective; nonetheless, even if this sign is subtle, a skilled and knowledgeable radiologist may be able to identify this sign in both MRI and CT scans.^31^, ^33^, ^34^

While posterior globe flattening is rarely observed in cases of ocular hypotony, it is more likely to indicate the presence of intracranial hypertension.^35^ This condition is believed to be a result of heightened CSF pressure that is transmitted from the subarachnoid space, through the optic nerve sheath, and onto the posterior globe.^36^

Imaging studies have reported rates of posterior globe flattening between 45% and 81%.^17^, ^37^, ^38^, ^39^ Agid et al.^32^ showed that the compression of the posterior globe is the primary diagnostic indicator correlated with IIH in adults (sensitivity 43.5%, specificity 100%). This finding is consistent in pediatric IIH with a sensitivity of 56% and a specificity of 100%.^37^ However, more evidence from the literature should be considered to provide a nuanced picture of the current evidence. The presence of posterior globe flattening in children may indicate a concurrent rise in ICP and papilledema. Furthermore, a correlation between CSF pressure and posterior globe flattening can be established.^31^, ^39^

Other studies have corroborated the significance of posterior globe flattening in diagnosing IIH. For instance, Bidot et al.^40^ demonstrated that posterior globe flattening, along with optic nerve sheath distension, significantly correlates with IIH, emphasizing the importance of using a combination of signs to enhance diagnostic accuracy. Similarly, Degnan and Levy^36^ found that posterior globe flattening, when observed alongside other MRI findings like transverse sinus stenosis and ES, strongly supports the diagnosis of IIH. Therefore, while posterior globe flattening is a critical indicator, it is the combination of multiple MRI features that provides a finely nuanced and robust diagnostic picture for IIH.

## 5 OPTIC NERVE TORTUOSITY

**Figure 3:**
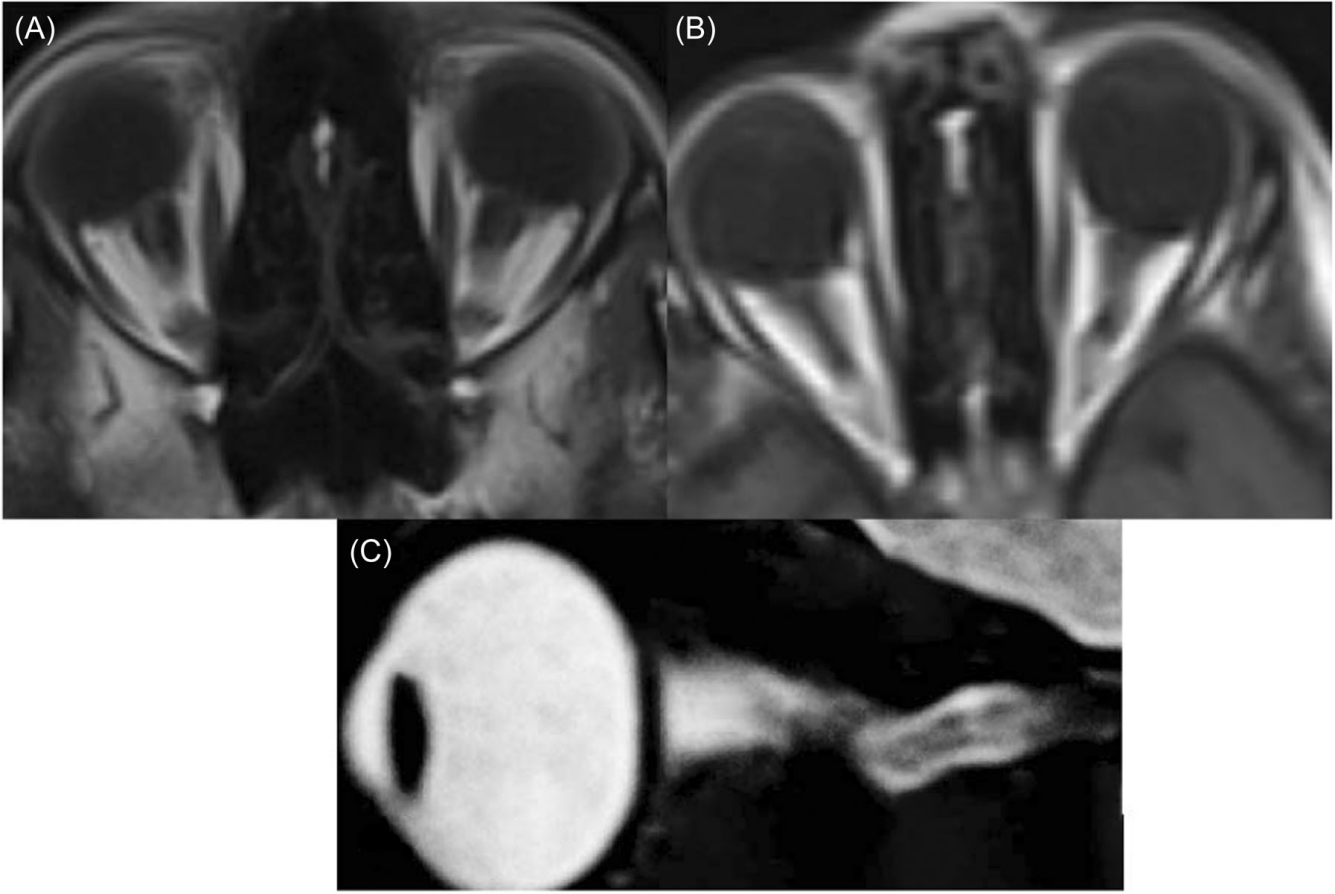
Optic nerve tortuosity. Loss of visualization of the middle portion of the orbital optic nerve and optic nerve tortuosity on T1‐weighted axial MRI is seen (A and B). An S‐shaped image of the optic nerve on sagittal images is seen (C). MRI, magnetic resonance imaging.

Whenever optic nerve tortuosity is incidentally detected on a brain MRI, it is always advisable to consider the possibility of optic nerve glioma, neurofibromatosis, or IIH.^32^, ^41^, ^42^ In the case of IIH, the optic nerve experiences tortuosity as a result of its fixation at both proximal and distal points, coupled with elevated CSF pressure within the optic nerve sheath.^43^, ^44^ When diagnosing optic nerve tortuosity, significant MRI findings to consider are the lack of congruity of the optic nerves on coronal sequences, the “smear sign” on T1‐weighted axial scans (showing loss of visualization of the middle portion of the orbital optic nerve), and the observation of an “S‐shaped” image of the nerve on sagittal images (Figure 3).^41^, ^42^, ^43^, ^45^

The optic nerve's flexible nature allows it to bend freely under pressure, resulting in horizontal or vertical tortuosity. The examination of horizontal tortuosity is favored due to its higher indication of intracranial hypertension, as found in the literature.^32^, ^37^, ^42^, ^44^ In the study conducted by Görkem et al.,^37^ horizontal optic nerve tortuosity showed a sensitivity of 68% and a specificity of 83%. Our review of the studies revealed that optic nerve tortuosity may be present in 40%–90.9% of pediatric patients, which is a higher proportion than the 35%–63% reported in the literature for adults.^17^, ^25^, ^31^, ^32^, ^45^, ^46^, ^47^

However, it is important to note that recent studies have questioned the sensitivity of optic nerve tortuosity for IIH, particularly in adults. For instance, a recently published literature showed that the sensitivity of optic nerve tortuosity for diagnosis of IIH was 26.5%.^48^ This suggests that while optic nerve tortuosity can be an indicator of IIH, its diagnostic value may vary, and it should be considered alongside other clinical and imaging findings.

## 6 OPTIC NERVE SHEATH DISTENSION

Due to its inherent nature, the optic nerve sheath exhibits relative flexibility, thus responding to changes such as enlargement caused by increased pressure.^49^, ^50^ The concept of an enlarged optic nerve sheath diameter (ONSD) as an indicator of ICP has developed over the past 25 years. This concept is justified based on the anatomical extension of the subarachnoid space beneath the optic nerve sheath and its association with cerebral CSF cavities. The belief is that increased ICP leads to the transmission of force through these spaces, which in turn causes distention of the ONSD. Additional evidence supporting this theory comes from studies demonstrating a similar correlation in the opposite direction (i.e., a small ONSD value when ICP is decreased).^51^, ^52^, ^53^

**Figure 4:**
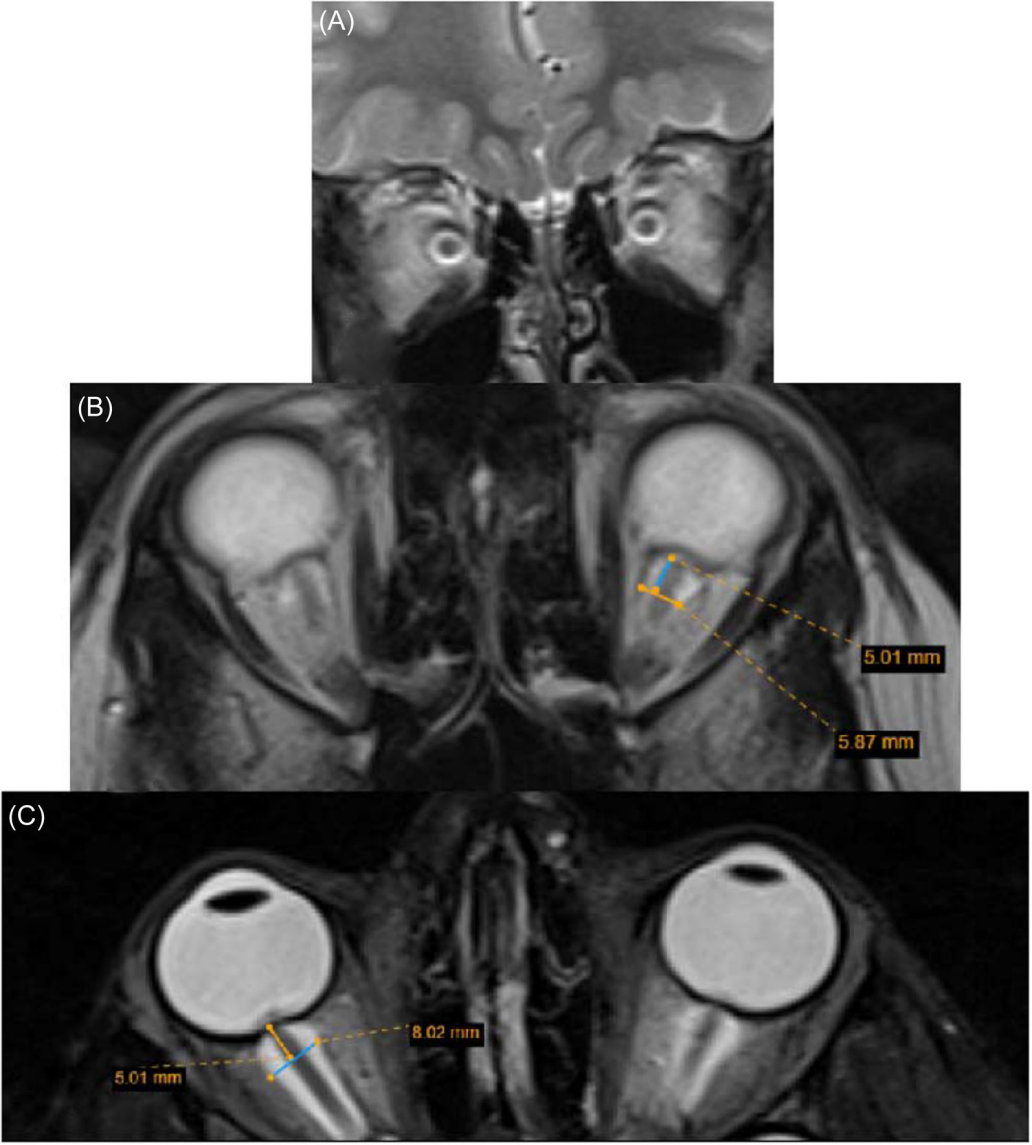
Optic nerve sheath distension. Expanded CSF signal intensity surrounding the optic nerve on T2‐ weighted coronal image (A). The optic nerve sheath diameter is measured at a point 5 mm posterior to the globe on an axial T2‐ weighted without (B) and with (C) fat suppression sequences. CSF, cerebrospinal fluid.

In IIH cases, the optic nerve sheath enlargement can be detected in T2 coronal images, presenting as an expanded CSF signal intensity surrounding the optic nerve.^31^ The measurement of the ONSD should be taken at a point 5 mm posterior to the globe on an axial T2‐weighted image.^25^, ^31^, ^37^ The addition of fat suppression makes this measurement easier but is not mandatory (Figure 4).^51^

Depending on the individual's age, the cutoff for ONSD is different, and it is essential to incorporate age‐adjusted norms to utilize ONSD in clinical practice accurately.^51^ According to the findings of Görkem et al.'s^37^ study, the optic nerve sheath width exhibited a specificity of 80% and a sensitivity of 88% when utilizing a cutoff value of 3.61 mm. Within the pediatric population, for children younger than 1 year of age, the upper limit is determined as 4.0 mm, while for older children, it is set at 4.5 mm.^54^, ^55^

A notable correlation between the ONSD and CSF opening pressure was observed in the study conducted by Kılıç et al.^24^ However, Hirfanoğlu et al.^31^ did not find any significant correlations between the CSF opening pressure and enlargement of the optic nerve sheath. This difference may be due to these studies’ small number of patients.

## 7 TRANSVERSE VENOUS SINUS STENOSIS

Stenosis of the transverse sinuses, which are venous channels found between the layers of the dura mater, has been linked to IIH.^36^, ^44^ The transverse sinuses in individuals diagnosed with IIH may show susceptibility to stenosis. Nonetheless, the ambiguity remains about whether this finding results from elevated ICP or is a fundamental component of the disease's pathophysiology.^56^, ^57^

**Figure 5:**
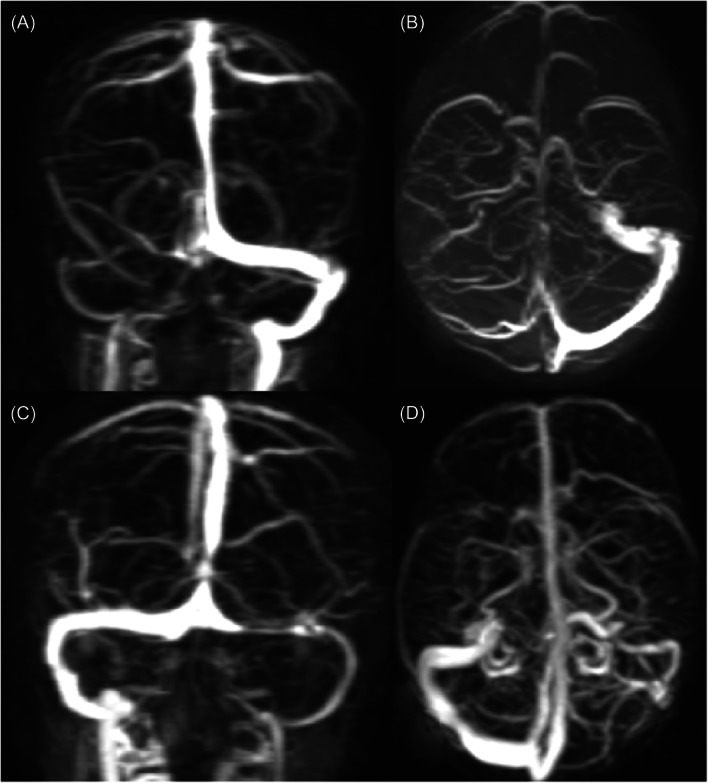
Non‐contrast‐enhanced MRV demonstrates stenosis of the right transverse sinus (A and B). Gadolinium‐enhanced MRV shows stenosis of the left transverse sinus (C and D).

The determination of transverse venous sinus stenosis can be made through the examination of maximum‐intensity projection images obtained from either a two‐dimensional time‐of‐flight MRV or contrast‐enhanced MRV.^15^ However, the detection of regions with subtle cerebral venous stenosis is better achieved through ^17^ the use of a gadolinium‐enhanced MRV in comparison to conventional MRV. However, the detection of regions with subtle cerebral venous stenosis is better achieved through the use of a gadolinium‐enhanced MRV in comparison to conventional MRV (Figure 5).^58^

The definition of transverse sinus stenosis involves observing sinus contour collapse or flattening, caliber alteration, or narrowing from the torcula to the distal sigmoid sinus.^58^, ^59^

In the study conducted by Gilbert et al.,^57^ it was found that dural venous sinus abnormalities, such as focal or diffuse long‐segment sinus narrowing, sinus atresia or absence, and the presence of a flow gap within the transverse sinus, were significantly correlated with IIH in the pediatric population.

In a separate study, it was observed that prepubescent children (<11 years) exhibited a lower incidence of transverse venous sinus stenosis (67%) compared to adolescents (11–17 years) and adults (>17 years) who had a higher frequency of transverse venous sinus stenosis (93% and 96%, respectively).^17^

It is important to note that a defined threshold for when stenosis is considered relevant for IIH has not been established. The lack of clear diagnostic criteria for the extent of stenosis necessary to impact IIH diagnosis emphasizes the need for further research to develop standardized thresholds.

## 8 OTHER DEBATABLE POINTS

Increased Meckel's cave length, cerebellar tonsillar herniation, and slit‐like ventricles are among the subtle imaging markers observed in some cases of pediatric IIH. However, these markers are not regarded as dependable indicators.^17^, ^25^, ^60^

The study analyzing diffusion‐weighted imaging findings of brain parenchyma in pediatric IIH did not identify any changes in cerebral ADC values that may suggest the presence of cerebral edema.^61^

The etiology of IIH remains incompletely comprehended, and IIH can manifest in the absence of papilledema^2^, ^9^; hence, MRI findings can serve as a valuable cautionary and non‐invasive diagnostic indicator for IIH. Moreover, implementing such findings can aid in identifying IIH and assist in clinical management and surgical intervention during the initial phases of IIH and papilledema.

Table 1 shows the summary of MRI finding in pediatric IIH.

**Table 1:** Summary of MRI findings in pediatric IIH.

MRI finding	Frequency (%)	Sensitivity	Specificity	Diagnostic value
Empty sella	25.9–78	Low	High	Limited
Posterior globe flattening	45–81	Moderate	Moderate to high	Important
Optic nerve tortuosity	40–90.9	Low	Moderate to high	Varies
Optic nerve sheath distension	55–88	Moderate to high	Moderate	Important
Transverse venous sinus stenosis	67–96	Varies	Varies	Important

Abbreviations: IIH, idiopathic intracranial hypertension; MRI, magnetic resonance imaging.

## 9 CONCLUSION

The presence of ES turcica, posterior globe flattening, optic nerve tortuosity, optic nerve sheath distension, and transverse venous sinus stenosis on MRI scans serve as crucial neuroradiological markers for diagnosing IIH in pediatric patients. These findings underscore the importance of MRI in clinical evaluation and management of pediatric IIH. Moving forward, future studies should aim to provide comprehensive explanations of these findings or explore novel imaging features specific to pediatric IIH. By enhancing our understanding of these MRI markers, we can improve diagnostic accuracy, guide therapeutic strategies, and ultimately optimize outcomes for pediatric patients with IIH.

## AUTHOR CONTRIBUTIONS

**Abdolreza Sheibani**: Writing—review and editing; methodology. **Narges Hashemi**: Writing—original draft; data curation; conceptualization; investigation; project administration; validation. **Behnam Beizaei**: Writing—original draft; writing—review and editing; data curation. **Nahid Tavakkolizadeh**: Writing—original draft; writing—review and editing; data curation. **Ahmad Shoja**: Writing—original draft; writing—review and editing; data curation; Methodology. **Neda Karimabadi**: Writing—original draft; writing—review and editing; data curation. **Houshang Mirakhorli**: Writing—original draft; writing—review and editing. **Parsa Hasanabadi**: Writing—review and editing. **Asma Payandeh**: Writing—original draft; writing—review and editing; data curation; methodology. **Ehsan Hassannejad**: Writing—original draft; writing—review and editing; data curation; methodology; visualization; investigation; conceptualization; project administration; supervision.

## CONFLICT OF INTEREST STATEMENT

The authors declare no conflicts of interest.

## ETHICS STATEMENT

All figures are of our patients. The confidentiality of the patient's information is maintained, and personal information, such as names and surnames, is not entered into the article. The figures are extracted from our study approved by the Mashhad University of Medical Sciences ethical committee with the ethics committee number 1398.085.IR.MUMS.MEDICAL.REC.

## TRANSPARENCY STATEMENT

The lead author Ehsan Hassannejad affirms that this manuscript is an honest, accurate, and transparent account of the study being reported; that no important aspects of the study have been omitted; and that any discrepancies from the study as planned (and, if relevant, registered) have been explained.

## Data Availability

The data that support the findings of this study are available on request from the corresponding author. The data are not publicly available due to privacy or ethical restrictions. The datasets created during the current study are not publicly accessible due to the possibility of compromising the privacy of individuals.
